# Effects of Cinnamon (*Cinnamomum cassia*) Consumption on Serum Lipid Profiles in Albino Rats

**DOI:** 10.1155/2020/8469830

**Published:** 2020-01-23

**Authors:** Fahadah Naeef Alsoodeeri, Hissah Mohammed Alqabbani, Norah Mubarak Aldossari

**Affiliations:** ^1^Department of Home Economics, College of Home Economics, King Khalid University, Abha, Saudi Arabia; ^2^Department of Home Economics, College of Education—Dilam, Prince Sattam Bin Abdulaziz University, Al-Kharj, Saudi Arabia; ^3^Department of Home Economics, College of Education—Wadi Addawasir, Prince Sattam Bin Abdulaziz University, Al-Kharj, Saudi Arabia

## Abstract

Dyslipidemia is an important cause of cardiovascular diseases (CVDs), which are the most prevalent causes of morbidity and mortality. The purpose of this study was to assess the effects of cinnamon on body weight gain, food intake, and serum lipid profiles of albino rats. This study was conducted on 30 healthy male albino rats weighing approximately 130 ± 5 g. The study was divided into the following two experiments: experiment (1), wherein rats were fed a laboratory diet; and experiment (2), wherein rats were fed a high-fat diet. In experiment 1, a total of 15 rats were divided into three groups. Group A (*n* = 5, untreated control) was fed laboratory diet, Group B (*n* = 5) was fed laboratory diet and cinnamon powder (2 g/kg body weight), and Group C (*n* = 5) was fed laboratory diet and cinnamon powder (4 g/kg body weight) for 30 days. In experiment (2), a total of 15 rats were similarly divided into three groups. Group D (*n* = 5, treated control) was fed laboratory diet plus high-fat diet, Group E (*n* = 5) was fed cinnamon powder (2 g/kg body weight) mixed with laboratory diet plus high-fat diet, and Group F (*n* = 5) was fed cinnamon powder (4 g/kg body weight) mixed with laboratory diet plus high-fat diet daily for 30 days. An administration of 4 g/kg body weight of cinnamon extract powder decreased the final weight by 4.4%, body weight gains by 31.41%, food intake by 1.7%, and food efficiency ratio by 22.38% in hypercholesterolemic adult male rats as well as serum total cholesterol by 31.22%, triglyceride by 24.05%, and LDL-C by 43.49%, with an increase in the levels of HDL-C by 30.16%, furthermore, a significant decrease in serum total cholesterol, triglycerides, and LDL-C levels and increasing serum HDL-C on day 30 were observed (*P* < 0.001). This finding provides scientific evidence to substantiate the traditional use of cinnamon to treat hyperlipidemia.

## 1. Introduction

Dyslipidemia is an important cause of cardiovascular diseases (CVDs), which are the most prevalent reasons for morbidity and mortality. These diseases are characterized by elevated blood lipids, which include at least one of the following alterations in the lipid profile: increased serum levels of low-density lipoprotein cholesterol (LDL-C) and triglycerides, and/or decreased levels of high-density lipoprotein cholesterol (HDL-C) in systemic circulation. These factors are among the leading causes of CVDs [1–3]. Globally, CVDs resulted in 17.3 million human deaths (32.1%) in 2015, an increase from the 12.3 million deaths (25.8%) reported in 1990; CVDs are responsible for over 31% of all deaths reported worldwide [4, 5]. In developing countries, mortality caused by CVDs is expected to rise to 19 million by 2020 [6].

The organic material cinnamon, one of the most important and popular spices used daily by people worldwide without any side effects, is extracted from the inner bark of trees of the genus *Cinnamomum* of the family Lauraceae. It is represented by approximately 250 species widely found across Asia, Australia, and South America [7, 8]. *Cinnamomum cassia*, called Chinese cassia or Chinese cinnamon is an evergreen tree originating in southern China, and widely cultivated there and elsewhere in southern and eastern Asia. It is one of the most important spices and medicinal materials in the world [9]. Cinnamon extract is a more concentrated source of flavor than ground cinnamon, similar to vanilla extract. Cinnamon primarily contains vital oils and other derivatives, such as cinnamaldehyde, cinnamic acid, and cinnamate, and these derivatives play vital roles in its natural antioxidant [10], anti-inflammatory [11], antidiabetic [12, 13], antimicrobial [14, 15], anticancer [16, 17], and cholesterol-lipid-lowering properties [12, 18, 19].

Previously, Kim et al. investigated the anti-diabetic effect of *Cinnamomi cassiae* extract at different dosages (50, 100, 150, and 200 mg/kg) in a mouse (C57BI/KsJ db/db) model of type II diabetes for six weeks. The administration of cinnamon to mice positively affected the lipid profile, whereby the serum HDL-C and triglyceride levels were reduced [20]. Another study reported that *C. cassiae* powder (15%) decreased serum total cholesterol, triglycerides, and LDL-C in rats after 35 days [21]. Recently, a study also reported that 500 mg of cinnamon water-extract used for two months by 137 men and women from Beijing and Dalian, China resulted in reduction of the total cholesterol and LDL-C levels [19]. However, the effect of cinnamon on blood lipids is still controversial and the mechanism by which cinnamon supplements influence the level of blood lipids needs further investigation. The present study aimed to assess the effects of cinnamon on body weight gain, food intake, food efficiency ratio, and serum lipid profile of albino rats.

## 2. Materials and Methods

This experimental study was conducted at the Department of Food Science and Nutrition, College of Food and Agriculture Sciences, King Saud University, Saudi Arabia from January 2017 to August 2017.

### 2.1. Sample, Diets, Chemicals, and Biochemical Analysis

Cinnamon bark (*Cinnamomum cassia*), cholesterol, and cholic acid were purchased from the local market in Riyadh, Saudi Arabia. Cinnamon extract was ground with a plant tissue grinder. Cinnamon bark (1 kg) was extracted twice with 640 ml of water for 16 h at 90°C and filtered. The water extract was lyophilized in a drying oven at 60°C, finely powdered with a mechanical mixer and then stored at room temperature until further use [20]. The finely lyophilized cinnamon was weighed (1 kg) and mixed with 1000 ml of water. This extract was diluted weekly with water and orally administered to the rats at particular doses. The cinnamon powder was then fed at a daily dose of 2 g/kg to the animals of treatment groups B, C, E, and F for 30 days. These four groups also received daily doses of cinnamon extract (4 g/kg body weight) for 30 days.

Total cholesterol, triglycerides, and HDL-C were purchased from Thermo Fisher (catalog number 981301 for triglycerides, 981812 for total cholesterol, and 981823 for HDL-C). Serum lipid profiles (total cholesterol, triglycerides, LDL-C, and HDL-C) were determined by the enzymatic colorimetric (CHOD-PAP) method using an automated biochemistry analyzer (Konelab 20, Thermo Fisher Scientific, Helsinki, Finland). Low density lipoprotein cholesterol (LDL-C) was estimated using the Friedewald formula [22].

### 2.2. Animals

Thirty healthy adult male albino rats (*Rattus norvegicus*) weighing approximately 130 ± 5 g were obtained from the animal rearing facility of the Zoology Department, College of Science, King Saud University, Saudi Arabia. Before the experiment, rats were fed water and laboratory diet (40 g/day) for 5 days and housed at 25 ± 5 °C under good ventilation. Ethical approval was obtained from the Ethics Committee of the College of Science Research Center of King Saud University, Riyadh, Saudi Arabia (8/25/256345).

### 2.3. Measurement of Growth Indicators

The body weight and food intake of the animals were recorded at the beginning of and during the experimental period.

#### 2.3.1. Body Weight Gain (g)

Weight gain for the entire 30-day period was calculated as final weight—initial weight for each rat.

#### 2.3.2. Food Intake (g)

Food intake was calculated every day as total food consumption/duration for each rat, all unconsumed food and pellets were removed and replaced with new laboratory diet (40 g/day).

#### 2.3.3. Food Efficiency Ratio

The food efficiency ratio was calculated as mean daily body weight gain (g)/mean daily food intake (g) for each rat.

### 2.4. Experiment Design 1

Experiment 1 was conducted to demonstrate the effect of cinnamon on normal adult male albino rats. A total of 15 rats were divided into three groups of 5 rats each. Each cage contained one rat and was labeled carefully for identification of the different groups.

*Group A (Untreated Control).* The group A rats served as untreated controls or normal controls, each albino rat received laboratory diet (40 g/day) and water for 30 days.

*Group B.* Each rat received cinnamon powder (2 g/kg body weight) mixed with laboratory diet (40 g/day) and water for 30 days.

*Group C.* Each rat received cinnamon powder (4 g/kg body weight) mixed with laboratory diet (40 g/day) and water for 30 days.

*Experiment Design 2.* Experiment 2 was conducted to demonstrate the effects of cinnamon on hypercholesterolemic rats.

*Group D (Treated Control).* Each rat served as a treated control and was orally administered laboratory diet (40 g/day) plus 1% cholesterol with 0.25% cholic acid and water for 30 days.

*Group E.* Each rat orally received cinnamon powder (2 g/kg body weight) mixed with laboratory diet (40 g/day) plus 1% cholesterol with 0.25% cholic acid and water for 30 days.

*Group F.* Each rat orally received cinnamon powder (4 g/kg body weight) mixed with laboratory diet (40 g/day) plus 1% cholesterol with 0.25% cholic acid and water for 30 days.

### 2.5. Collection of Blood Specimens

At the end of 30 days, after a 12 h fasting period, all rats were euthanized using diethyl ether as the anesthetic agent. Blood samples were collected in test tubes and allowed to coagulate at room temperature. Each sample was transferred into a dry clean centrifuge tube and centrifuged at 3000 rpm for 30 min for separating serum. The supernatant sera were quickly removed and stored at −20 °C for biochemical analysis of serum lipid profiles.

### 2.6. Statistical Analysis

SPSS version 16 (SPSS Inc. Chicago, IL, USA) was used for data acquisition and analysis. Data are presented as mean and standard deviation (SD) for continuous variables. Frequencies are presented as percentages (%). Comparisons between groups were conducted using independent *t*-tests for continuous variables and Chi-square tests for categorical variables. To elucidate the effects of cinnamon on the biochemical parameters and body weight of the rats, paired *t*-tests were conducted. Statistical significance was set at *P* < 0.05.

## 3. Results

### 3.1. Experiment 1

The effects of the cinnamon extract powder on body weight gain, food intake, food efficiency ratio, and serum lipid profiles of normal adult male rats are shown in Table 1. On day 30 post-treatment, the cinnamon extract powder equivalent to 2 g/kg body weight reduced the final weight by 3.22%, body weight gains by 13.98%, food intake by 3.59%, and food efficiency ratio by 10.71%. With respect to the lipid profile, serum total cholesterol was reduced by 2.10%, triglycerides by 3.65%, and LDL-C by 10.37%. Although an increase was observed in HDL-C by 3.83%, it was not a significant change. The administration of 4 g/kg of body weight of cinnamon extract powder also reduced the final weight by 2.59%, body weight gains by 19.00%, food intake by 4.31%, food efficiency ratio by 15.47%, serum total cholesterol by 4.1%, triglycerides by 4.87%, and LDL-C levels by 10.28%. The level of HDL-C was increased by 7.08%. However, the changes in these parameters were non-significant.

### 3.2. Experiment 2

Comparison of weight, food intake, and lipid profile of adult, male rats fed normal laboratory diet (Group A) and high-cholesterol diet (Group D) is shown in Table 2. Final weight, body weight gain, and food intake values of the treated control (Group D) were significantly increased compared to those of the untreated control group (Group A) (*P* < 0.001). Serum total cholesterol, triglyceride, and LDL-C levels in the treated control (Group D) were also significantly increased than those of the untreated control (Group A) (*P* < 0.001).

The effects of cinnamon extract powder on body weight gain, food intake, food efficiency ratio, and serum lipid profiles of hypercholesterolemic adult male rats are shown in Table 3. Cinnamon extract powder equivalent to 2 g/kg body weight decreased final weight by 3.11%, body weight gains by 23.94%, food intake by 3.31%, and food efficiency ratio by 13.43%. The lipid profile parameters also showed significant reduction; serum total cholesterol by 27.32%, triglycerides by 16.65%, and LDL-C by 49.06%, with an increase in levels of HDL-C by 25.56% after 30 days of treatment. However, we did not observe a significant change. An administration of 4 g/kg body weight of cinnamon extract powder decreased the final weight by 4.4%, body weight gain by 31.41%, food intake by 1.7%, and food efficiency ratio by 22.38%. In addition, serum total cholesterol was reduced by 31.22%, triglycerides by 24.05%, and LDL-C by 43.49%, with an increase in the levels of HDL-C by 30.16%. Significant reductions were recorded only for body weight gain, serum total cholesterol, triglycerides, and LDL-C (*P* < 0.001).

The differences between Group E (2 g/kg cinnamon extract powder) and Group F (4 g/kg cinnamon extract powder) in hypercholesterolemic adult male rats are shown in Table 4. Serum total cholesterol and triglycerides in Group E were significantly higher than those in Group F, whereas LDL-C was significantly lower (*P* < 0.001). No other statistically significant differences were observed.

The comparison of effects of cinnamon extract powder on body weight gain, food intake, food efficiency, and serum lipid profile in normal and hypercholesterolemic adult male rats Group B vs Group E and Group C vs Group F is shown in Table 5. The final weight and food intake as well as serum total cholesterol, triglycerides, and LDL-C in Group B were significantly lower than those in Group E (*P* < 0.001). Similarly, food intake, serum total cholesterol, triglycerides, and LDL-C in Group C were significantly reduced compared to those in Group F (*P* < 0.001). No other statistically significant differences were observed.

## 4. Discussion

Based on the results, a significant increase in the final weight, body weight gain, and food intake was observed (Table 2) (*P* < 0.001) after administration of the high-fat mixture (1% cholesterol with 0.25% cholic acid) for 30 days in the treated control (Group D) compared to that of the untreated control (Group A). This was possibly caused by the accumulation of fat in the body. These findings are consistent with the results of a previous study which showed a rapid increase in the body weight of rats fed a high-fat diet over 8 weeks [23]. In contrast, another study did not support these results [24].

The serum total cholesterol, triglyceride, and LDL-C levels were also significantly increased after the 30-day administration of the high-fat mixture (Table 2) (*P* < 0.001). These findings were consistent with those of several previous studies, which showed that rats fed a diet rich in cholesterol had increased serum lipid profile parameters [21, 25–27]. Cholic acid increases hyperlipidemia possibly through two mechanisms, an increase in cholesterol absorption and a concomitant suppression of cholesterol 7*α*-hydroxylase activity that results in decreased cholesterol excretion. Cholic acid supplementation also enhances cholesterol absorption by its emulsifying property [28].

The bark of various cinnamon species is used worldwide as a spice in cooking. Cinnamon has been considered a safe flavoring agent in food for thousands of years, and there have been no reports of any side effects [29, 30]. We observed a slight decrease in the final weight, body weight gain, food intake, as well as serum total cholesterol, triglycerides, and LDL-C in normal adult male rats treated with cinnamon extract powder (Group B and Group C) compared to those in the untreated control (Group A). However, these differences were not significant (Table 1). These observations were consistent with the results of previous studies [21, 23, 31].

The most important finding of this study was that cinnamon extract powder at doses of 2 g/kg body weight per day for 30 days slightly reduced body weight gain, serum total cholesterol, triglycerides, and LDL-C levels, whereas HDL-C was increased compared to those of the treated control (Group D) in hyperlipidemic adult male rats (Table 3). These results are consistent with previous studies reporting that cinnamon powder at 2 g/kg body weight decreased the serum total lipids by 11.83%, total cholesterol by 31.58%, triglycerides by 33.50%, and LDL-C by 63.40%, whereas HDL-C was increased by 56.36% on day 60 post treatment [26]. Rahman et al. also reported that 15% cinnamon powder significantly decreased the serum total cholesterol by 12%, triglycerides by 11%, LDL-C by 14% in high-fat mixture-fed rats [21]. Similar findings were obtained in another study [32]. Our study showed that the administration of 4 g/kg body weight of cinnamon extract powder significantly reduced the levels of the serum lipid profile (total cholesterol, triglycerides, and LDL-C) (Table 3) (*P* < 0.001). This is in accordance with a previous finding by Iqbal et al., who showed that administration of a high-cholesterol diet with cinnamon powder equivalent to 4 g/kg body weight significantly decreased the serum total lipids, total cholesterol, triglycerides, and LDL-C in rats on day 60 post-treatment [26].

Cinnamon extract improves hyperlipidemia, possibly by playing a direct role in lipid metabolism by inhibiting hepatic *β*-hydroxy *β*-methylglutaryl-CoA (HMG-CoA) reductase activity, resulting in lower cholesterol production in the liver and suppression of lipid peroxidation [33]. Furthermore, increased lecithin cholesterol acyl transferase (LCAT) activity, which is essential for blood lipid regulation, can cause elevated HDL-C levels [34, 35]. The decreased triglyceride levels may be caused by the lipolytic action of cinnamon. In addition, the inhibition of triglyceride synthesis may also be responsible for maintenance of a low level of triglycerides [36].

However, the present study has some limitations. *Cinnamomum cassia* extract is effective in preventing hyperlipidemia, the most important risk factor associated with high incidence of myocardial infarctions and CVDs. Therefore, future studies should focus on identifying the major components responsible for hyperlipidemia and also aim to elucidate its underlying mechanism. Further studies could also establish the effect of *Cinnamomum cassia* on hyperlipidemic patients.

## 5. Conclusion

In conclusion, the results of the present study revealed that cinnamon extract has a hypolipidemic effect on hypercholesterolemic albino rats at different doses. Daily administration of cinnamon extract equivalent to 4 g/kg showed significant antihyperlipidemic effect in hypercholesterolemic albino rats by decreasing serum total cholesterol, triglyceride, and LDL-C levels and increasing serum HDL-C on day 30 (*P* < 0.001). The mechanisms underlying the hypolipidemic effect and its cellular mechanism of action need to be elucidated in future studies.

## Figures and Tables

**Table 1:** Effects of cinnamon extract powder on body weight gain, food intake, food efficiency ratio, and serum lipid profiles of normal adult male rats.

Parameters	Group A	Group B	Group C	Percentage reduction			
	(*n* = 5)	(*n* = 5)	(*n* = 5)	Group B		Group C	
				%	*P* value	%	*P* value
Initial weight (g)	131.8 ± 1.2	130.12 ± 2.3	132.3 ± 2.1	1.3	0.62	0.33	0.56
Final weight (g)	155.4 ± 3.	150.4 ± 3.6	151.3 ± 3.6	3.2	0.35	2.59	0.12
Body weight gain (g)	23.5 ± 2.1	20.2 ± 1.4	19.1 ± 1.4	13.9	0.29	19.00	0.32
Food intake (g)	27.8 ± 6.6	26.8 ± 6.1	26.6 ± 5.3	3.6	0.63	4.31	0.56
Food efficiency ratio	0.84 ± 0.31	0.75 ± 0.22	0.71 ± 0.26	10.71	0.69	15.47	0.36
Serum total cholesterol (mg/dL)	95.1 ± 2.5	91.2 ± 1.5	92.1 ± 2.5	2.1	0.45	4.14	0.12
Serum triglycerides (mg/dL)	82.1 ± 2.5	79.1 ± 2.5	78.1 ± 2.4	3.7	0.36	4.87	0.25
Serum HDL-C (mg/dL)	33.9 ± 0.7	35.2 ± 0.8	36.3 ± 0.7	3.8	0.24	7.08	0.35
Serum LDL-C (mg/dL)	44.8 ± 1.3	40.2 ± 1.8	40.2 ± 1.2	10.4	0.36	10.28	0.35

Data presented are mean ± SD. *P*-value significant at <0.05.

**Table 2:** Weight, food intake and lipid profiles for adult, male rats on normal laboratory diet (Group A) compared to a high cholesterol diet (Group D).

Parameters	Group A	Group D	*P* value
	(*n* = 5)	(*n* = 5)	
Initial weight (g)	131.8 ± 1.2	133.1 ± 2.5	0.28
Final weight (g)	155.4 ± 3.	159.3 ± 4.3	<0.001
Body weight gain (g)	23.5 ± 2.1	29.2 ± 1.8	<0.001
Food intake (g)	27.8 ± 6.6	39.2 ± 8.5	<0.001
Food efficiency ratio	0.84 ± 0.31	0.67 ± 0.21	0.06
Serum total cholesterol (mg/dL)	95.1 ± 2.5	138.3 ± 5.9	<0.001
Serum triglycerides (mg/dL)	82.1 ± 2.5	108.1 ± 2.4	<0.001
Serum HDL-C (mg/dL)	33.9 ± 0.7	30.9 ± 0.8	0.07
Serum LDL-C (mg/dL)	44.8 ± 1.3	80.8 ± 4.6	<0.001

Data presented are mean ± SD. *P*-value significant at <0.05.

**Table 3:** Effects of cinnamon extract powder on body weight gain, food intake, food efficiency, and serum lipid profiles in hypercholesterolemic adult male rats.

Parameters	Group D	Group E	Group F	Percentage change			
	(*n* = 5)	(*n* = 5)	(*n* = 5)	Group E		Group F	
				*%*	*P* value	%	*P* value
Initial weight (g)	133.1 ± 2.5	132.2 ± 3.2	132.3 ± 3.2	0.72	0.62	0.73	0.53
Final weight (g)	159.3 ± 4.3	154.4 ± 4.2	152.3 ± 5.3	3.11	0.58	4.4	0.35
Body weight gain (g)	29.2 ± 1.8	22.2 ± 1.0	20.0 ± 2.1	23.94	0.24	31.41	<0.001
Food intake (g)	39.2 ± 8.5	37.93 ± 6.1	38.6 ± 4.4	3.31	0.65	1.75	0.35
Food efficiency ratio	0.67 ± 0.21	0.58 ± 0.17	0.52 ± 0.49	13.43	0.45	22.38	0.35
Serum total cholesterol (mg/dL)	138.3 ± 5.9	100.5 ± 3.5	95.1 ± 4.4	27.32	0.25	31.22	<0.001
Serum triglycerides (mg/dL)	108.1 ± 2.4	90.1 ± 2.6	82.1 ± 3.5	16.65	0.36	24.05	<0.001
Serum HDL-C (mg/dL)	30.9 ± 0.8	38.8 ± 0.8	40.2 ± 0.6	25.56	0.65	30.16	<0.001
Serum LDL-C (mg/dL)	80.8 ± 4.6	43.7 ± 2.1	48.5 ± 3.1	49.06	0.48	43.49	<0.001

Data presented are mean ± SD. *P*-value significant at <0.05.

**Table 4:** Differences between Group E (2 g/kg cinnamon extract powder) and Group F (4 g/kg cinnamon extract powder) in hypercholesterolemic adult male rats (E vs F).

Parameters	Group E	Group F	*P* value
	(*n* = 5)	(*n* = 5)	
Initial weight (g)	132.2 ± 3.2	132.3 ± 3.2	0.53
Final weight (g)	154.4 ± 4.2	152.3 ± 5.3	0.29
Body weight gain (g)	22.2 ± 1.0	20.0 ± 2.1	0.42
Food intake (g)	37.93 ± 6.1	38.6 ± 4.4	0.21
Food efficiency ratio	0.58 ± 0.17	0.52 ± 0.49	0.62
Serum total cholesterol (mg/dL)	100.5 ± 3.5	95.1 ± 4.4	<0.001
Serum triglycerides (mg/dL)	90.1 ± 2.6	82.1 ± 3.5	<0.001
Serum HDL-C (mg/dL)	38.8 ± 0.8	40.2 ± 0.6	0.24
Serum LDL-C (mg/dL)	43.7 ± 2.1	48.5 ± 3.1	<0.001

Data presented are mean ± SD. *P*-value significant at <0.05.

**Table 5:** Comparison of effects of cinnamon extract powder on body weight gain, food intake, food efficiency, and serum lipid profiles in normal and hypercholesterolemic adult male rats Group (B vs E) and (C vs F).

Parameters	Group B	Group E	*P* value	Group C	Group F	*P* value
	(*n* = 5)	(*n* = 5)		(*n* = 5)	(*n* = 5)	
Initial weight (g)	130.12 ± 2.3	132.2 ± 3.2	0.62	132.3 ± 2.1	132.3 ± 3.2	0.98
Final weight (g)	150.4 ± 3.6	154.4 ± 4.2	<0.001	151.3 ± 3.6	152.3 ± 5.3	0.48
Body weight gain (g)	20.2 ± 1.4	22.2 ± 1.0	0.08	19.1 ± 1.4	20.0 ± 2.1	0.49
Food intake (g)	26.8 ± 6.1	37.93 ± 6.1	<0.001	26.6 ± 5.3	38.6 ± 4.4	<0.001
Food efficiency ratio	0.75 ± 0.22	0.58 ± 0.17	0.08	0.71 ± 0.26	0.52 ± 0.49	0.62
Serum total cholesterol (mg/dL)	91.2 ± 1.5	100.5 ± 3.5	<0.001	92.1 ± 2.5	95.1 ± 4.4	<0.001
Serum triglycerides (mg/dL)	79.1 ± 2.5	90.1 ± 2.6	<0.001	78.1 ± 2.4	82.1 ± 3.5	<0.001
Serum HDL-C (mg/dL)	35.2 ± 0.8	38.8 ± 0.8	0.24	36.3 ± 0.7	40.2 ± 0.6	0.06
Serum LDL-C (mg/dL)	40.2 ± 1.8	43.7 ± 2.1	<0.001	40.2 ± 1.2	48.5 ± 3.1	<0.001

Data presented are mean ± SD. *P*-value significant at <0.05.

## Data Availability

The data used to support the findings of this study are available from the corresponding author upon request.
